# Role of circRMRP and circRPL27 in chronic obstructive pulmonary disease

**DOI:** 10.1515/biol-2022-0942

**Published:** 2024-12-31

**Authors:** JianFang Li, PengFei Zhang, XianJing Zeng, Rong Liu

**Affiliations:** Department of General Practice, Wuhan Third Hospital Tongren Hospital of Wuhan University, Wuhan, Hubei, 430000, China; Department of Oncology, Jishui People’s Hospital, Ji’an, Jiangxi, 331600, China; Department of General Practice, Affiliated Hospital of Jinggangshan University, Ji’an, Jiangxi, 343000, China; Department of Respiratory and Critical Care Medicine, Huaian Hospital of Huaian City, No. 19 Shanyang Avenue, Huaian District, Huaian, Jiangsu, 223200, China

**Keywords:** chronic obstructive pulmonary disease, circRMRP, circRPL27, biomarkers

## Abstract

Chronic obstructive pulmonary disease (COPD) is one of the leading causes of death and disability worldwide, and circRNA dysregulation is functionally associated with COPD. This study explored the potential of circRMRP and circRPL27 as biomarkers of COPD. Blood samples from COPD patients and healthy controls were collected. COPD patients were divided into mild, moderate, and severe groups according to lung function. Quantitative real-time polymerase chain reaction technology was used to determine the expression of circRPL27 and circRMRP in COPD. Receiver operating characteristic curve was drawn to explore the value of circRMRP and circRPL27 in diagnosing COPD. circRMRP and circRPL27 levels were elevated in serum of COPD patients and increased with the severity of COPD. CircRMRP and circRPL27 were associated with smoking history, WBC, and FEV1/FVC, and were positively correlated with smoking history and WBC, and negatively correlated with FEV1/FVC. In COPD, both circRMRP and circRPL27 had diagnostic values, but circRPL27 was better. circRMRP and circRPL27 may be useful non-invasive biomarkers for COPD diagnosis.

## 1 Introduction

Globally, chronic obstructive pulmonary disease (COPD), a respiratory illness, ranks among the leading causes of death and disability [1,2]. COPD is estimated to become the third most common cause of death worldwide by 2030, influenced by several factors, such as an aging population [3–5]. More importantly, the COVID-19 pandemic has made routine management and diagnosis of COPD more difficult. Due to the similarity of symptoms between COVID-19 and COPD, it is also highly infectious, which brings great difficulties to the early diagnosis of these two diseases and affects the timely management and treatment of the diseases. In clinical settings, individuals suffering from severe COPD have a less favorable outlook than those with mild airflow restrictions. Compared to individuals with moderate or mild COPD, those suffering from severe COPD exhibit more obvious signs of discomfort, including pain and sleep disturbances [6]. A more efficient treatment approach can therefore be developed by differentiating key signs between individuals with severe COPD and healthy individuals.

Circular RNA (circRNA) is a non-coding RNA that is widely found in cells and remains relatively stable in plasma due to the absence of specific binding sites for nucleic acid exonucleases. circRNAs bind to miRNAs and competitively inhibit the activity of miRNAs to regulate gene expression. Many studies have shown aberrant expression of circRNAs in COPD patients. Guo et al. found that circRNA BBS9 may serve as a new target for the treatment of COPD [7]. Chen et al. identified a novel circRNA0001859, which may serve as a potential therapeutic biomarker for the treatment of COPD and acute exacerbation of COPD [8]. These evidence indicate that the levels of circRNAs in plasma of COPD patients are significantly changed, which may become a diagnostic marker of COPD or a new therapeutic intervention target [9,10]. Duan et al. analyzed circRNAs by microarray, and systematically compared the expression of circRNAs to screen out the most differentially expressed circRNAs in COPD [11]. Among them, we found that circ_0000157 (hsa_circSELL_002), circ_0043926 (hsa_circRPL27_001), and circRNA0001853 (hsa_circRMRP_001) were significantly up-regulated in COPD. Previous studies have shown that circSELL knockdown improves 16HBE cell damage by targeting the miR-149-5p/BRD4 pathway, providing a potential therapeutic strategy for clinical intervention in COPD [12]. However, the current in-depth study of circRMRP and circRPL27 is very limited. The potential value of circRMRP and circRPL27 in the clinical diagnosis of COPD remains to be studied.

In this study, blood samples from clinical COPD patients and healthy controls were collected to investigate the value of circRMRP and circRPL27 in COPD diagnosis and disease assessment. The findings suggest that circRMRP and circRPL27 may serve as diagnostic and therapeutic targets in COPD.

## 2 Materials and methods

### 2.1 Study population

Fifty COPD patients and 50 healthy controls who were hospitalized in Wuhan Third Hospital·Tongren Hospital of Wuhan University between January 2022 and January 2023 were selected to participate in this clinical study. According to the epidemiological survey results, the prevalence rate of COPD was 3.5–19.1%. The sample size was estimated according to the prevalence rate of 3.5%. At the test level of *α* = 0.05, the allowable error was limited to 2%. Taking into account factors such as 10% loss of the patients, a total of 100 subjects were finally included in the study, of which 50 were patients with COPD. Patients who complied with the guidelines for the diagnosis and treatment of COPD were included. COPD patients were categorized according to the Global Initiative for Chronic Obstructive Lung Disease (GOLD) criteria (2018): mild (GOLD grade 1), moderate (GOLD grade 2), severe (GOLD grade 3 and 4) [5]. Seven patients (14%) were mild, 16 patients (32%) were moderate, and 27 patients (54%) were severe. There were 41 males and 9 females with COPD, aged 45–78 years, with a mean age of (64 ± 4) years. Body mass index (BMI) ranged from 18.8 to 22.7 kg/m^2^ with a mean of 21.1 kg/m^2^. The patients had a smoking history of 0–32 years.

Inclusion criteria were (1) patients met the diagnosis of COPD in the guidelines of the Respiratory Division of the Chinese Medical Association and (2) patients were 18–90 years old.

Exclusion criteria included: (1) patients with restrictive ventilatory dysfunction, (2) patients with other chronic systemic and serious diseases that affect pulmonary function outcomes, (3) patients with a positive bronchodilator test result, and (4) patients who are unable to communicate and unwilling to cooperate.

Fifty healthy controls were also randomly selected, of which 39 were male and 11 were female, aged from 46 to 79 years, with a mean of (62 ± 5) years. BMI ranged from 18.4 to 21.6 kg/m^2^ with a mean of 20.7 kg/m^2^. The smoking history of the healthy individuals ranged from 0 to 24 years. There were no differences between sex ratio, BMI, and age between the COPD group and the healthy control group.


**Informed consent:** Informed consent has been obtained from all individuals included in this study.
**Ethical approval:** The research related to human use has been complied with all the relevant national regulations, institutional policies and in accordance with the tenets of the Helsinki Declaration, and has been approved by the Ethics Committee of Wuhan Third Hospital·Tongren Hospital of Wuhan.

### 2.2 Reverse transcription quantitative polymerase chain reaction (RT-qPCR) validation

#### 2.2.1 RNA extraction

About 3 mL of 24 h fasting venous blood was collected from all patients and healthy controls and frozen at −80°C. The sample was centrifuged at 4°C, 3,000 rpm for 15 min, and the supernatant was transferred into sterilized EP tubes and kept frozen at −80°C. Total RNA was extracted from the samples using TRIzol reagent (Invitrogen, USA). The RNA content of the samples was determined using a microspectrophotometer (Thermo, USA).

#### 2.2.2 cDNA synthesis

The cDNA of circRNA was synthesized using PrimeScript™ RT Reagent kit (TaKaRa, Japan). cDNA synthesized was mixed with SYBR ®Premix Ex Taq™ (Takara), and RT-qPCR reaction was carried out to detect circRMRP and circRPL27 levels in the ABI Prism 7300 system (Applied Biosystems, USA). The PCR amplification procedures were as follows: 40 cycles at 95°C for 10 min, followed by 90°C for 10 s, 60°C for 20 s, and 72°C for 30 s.

#### 2.2.3 qPCR quantification

The primers were synthesized by Beijing Genomics Institute (China) and the results were calculated by the 2^−ΔΔCT^ method. The primer sequences are shown in Table 1.

**Table 1:** Sequences of RT-qPCR primers

Name	Primer sequences (5′–3′)
circRMRP	Forward: AAAGGGGAGGAACAGAGTCC
	Reverse: TCATCCGTCAGCTCCCTCTA
circRPL27	Forward: GGCCAAGCATTGAGCATTAC
	Reverse: TGTTCCTCTGGCTCCCATAC
GAPDH	Forward: GGTGAAGGTCGGAGTCAAC
	Reverse: AGAGTTAAAAGCAGCCCTGGTG

### 2.3 Observations

The significance of circRMRP and circRPL27 in diagnosing COPD was assessed using receiver operating characteristic (ROC) curves. On ROC curves, the rate of positive results is displayed in vertical coordinates, and the rate of false positives is displayed in horizontal coordinates. The area under the curve (AUC) was calculated by the *Z*-test.

### 2.4 Statistical analysis

The data were statistically analyzed using SPSS 25.0 and plotted using GraphPad Prism 8.0 software. The Shapiro–Wilk test method was used for normality testing. Measures that conformed to the normal distribution were expressed as mean ± standard deviation. The *t*-test was taken to test the difference between two independent samples. The diagnostic value of circRMRP and circRPL27 was evaluated using the ROC curve. *P* < 0.05 was considered a statistically significant difference.

## 3 Results

### 3.1 Clinical data

There was no statistically significant difference between the COPD patient group and the control group in terms of male to female ratio, age, duration of COPD, length of hospitalization, and FEV1%; and there was a statistically significant difference between the COPD patient group and the control group in terms of smoking history, WBC, and FEV1/FVC level (Table 2).

**Table 2:** Comparison of clinical data

Indicators	Control group (*n* = 50)	COPD group (*n* = 50)	*P*-value
Male (%)	78	82	—
Age (years)	62 ± 5	64 ± 4	0.1218
BMI (body mass index)	20.7 ± 2.1	21.1 ± 2.3	0.5283
Smoking	14.7 ± 2.3	29.3 ± 5.7*	<0.001
Course of disease (years)	—	9.5 ± 1.2	—
Hospitalization (days)	—	11 ± 2	—
WBC (×10^9^/L)	8.3 ± 1.3	13.6 ± 3.1*	<0.001
FEV1/FVC (%)	77.9 ± 5.1	63.4 ± 3.8*	0.0418
FEV1% (%)	58.4 ± 4.6	57.7 ± 4.3*	0.6464

COPD: chronic obstructive pulmonary disease; WBC: white blood cells; FEV1/FEV: ratio of forced expiratory volume in 1 s to forced expiratory volume, which is a valid clinical indicator for determining the presence or absence of airflow limitation; FEV1%: refers to forced expiratory volume in 1 s. Note: *P < 0.05 vs control group.

### 3.2 circRMRP and circRPL27 expression in COPD group and healthy controls

**Figure 1:**
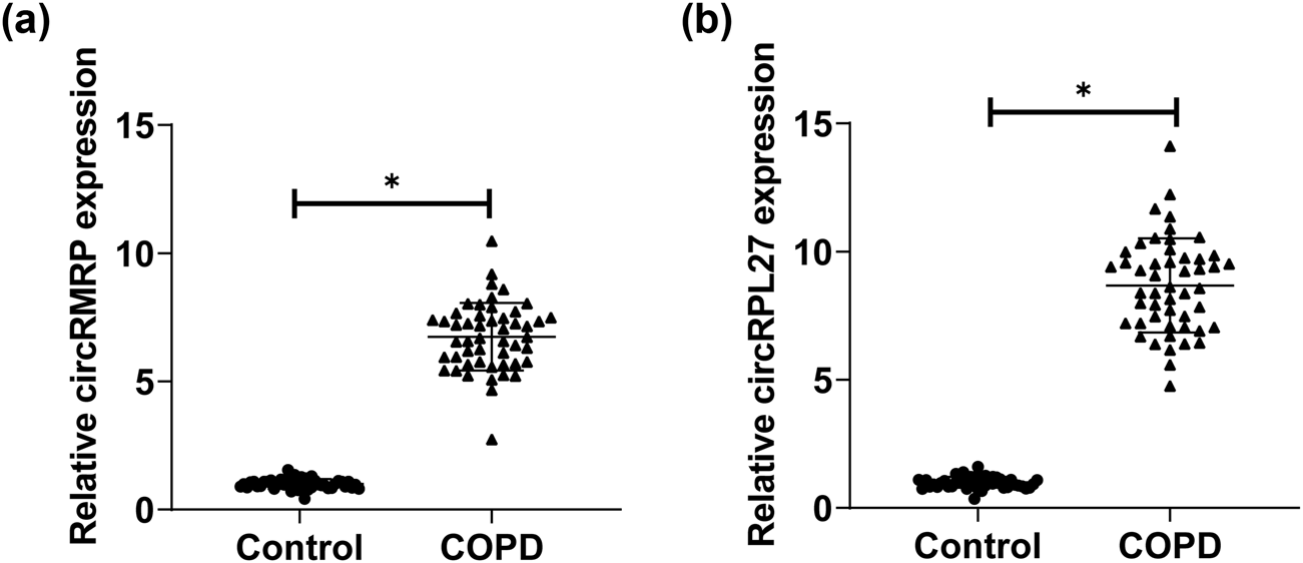
Comparison of the expression of circRMRP and circRPL27 in COPD group and healthy control group (*P < 0.05). (a) RT-qPCR to detect the expression level of circRMRP in the serum of COPD patients and (b) RT-qPCR to detect the expression level of circRPL27 in the serum of COPD patients.

circRMRP and circRPL27 levels were increased in patients in the COPD group (Figure 1).

### 3.3 Serum circRMRP and circRPL27 levels in the mild, moderate, and severe groups

The serum circRMRP and circRPL27 levels in the mild, moderate, and severe groups were higher than those in the healthy control group. Patients in the severe group showed higher circRMRP and circRPL27 than those in the moderate and mild groups. Patients in the moderate group expressed higher circRMRP and circRPL27 than those in the mild group (Table 3).

**Table 3:** Comparison of serum levels of circRMRP and circRPL27 in the three groups

Groups	Sample size (*n*)	circRMRP	circRPL27
Mild group	7	2.79 ± 0.46*	2.66 ± 0.54*
Moderate group	16	5.12 ± 1.31*	6.47 ± 1.09*
Severe group	27	8.54 ± 1.13*	9.84 ± 1.46*
Control group	50	1 ± 0.19	1 ± 0.21

Note: *P < 0.05 vs control group.

### 3.4 Correlation analysis between serum circRMRP and circRPL27 and other indicators in COPD patients

**Figure 2:**
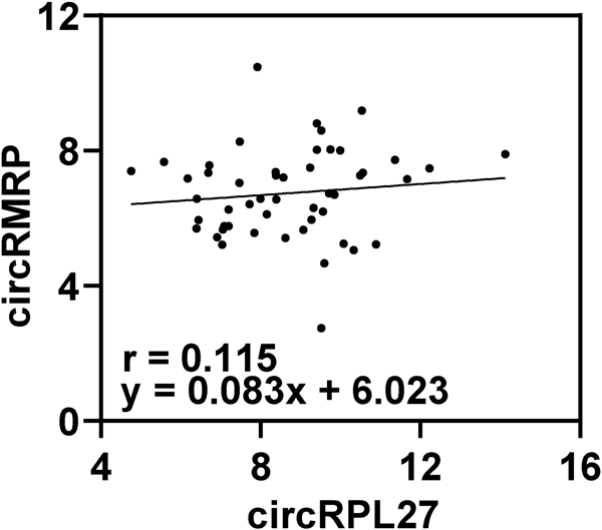
Correlation analysis of circRMRP and circRPL27 (*P* < 0.05).

Correlation analysis showed that circRMRP was positively correlated with smoking history and WBC, and negatively correlated with FEV1/FVC levels. circRPL27 was positively correlated with smoking history, WBC, and negatively correlated with FEV1/FVC and FEV1 levels (Table 4). In addition, as shown in Figure 2, circRPL27 and circRMRP were positively correlated.

**Table 4:** Correlation analysis between circRMRP and circRPL27 in serum and other indicators in COPD patients

Indicators	circRMRP		circRPL27	
	*r*	*P*	*r*	*P*
Smoking	0.31	<0.05	0.39	<0.05
Course of disease (years)	0.13	>0.05	0.26	>0.05
Hospitalization (days)	0.27	>0.05	0.24	>0.05
WBC (×10^9^/L)	0.31	<0.05	0.47	<0.05
FEV1/FEV (%)	−0.39	<0.05	−0.42	<0.05
FEV1% (%)	−0.33	>0.05	−0.34	<0.05

Note: *P < 0.05 vs control group.

### 3.5 Evaluation of the diagnostic value of serum circRMRP and circRPL27 expression

**Figure 3:**
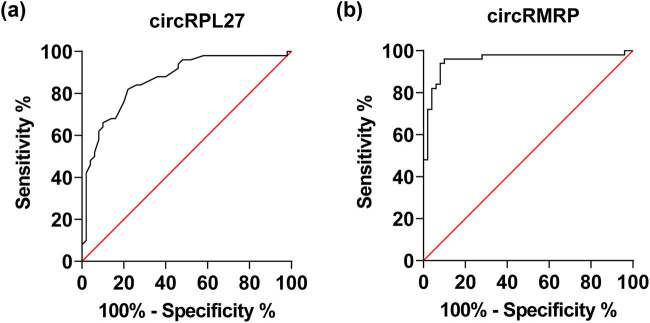
ROC curves of circRMRP and circRPL27 in serum for the diagnosis of COPD (*P* < 0.05). (a) ROC curve for circRPL27 and (b) ROC curve for circRMRP.

The ROC curves of circRMRP and circRPL27 in serum were plotted, and the AUC was calculated. The results showed that the AUC value of circRMRP in serum was 0.8646, and the AUC value of circRPL27 was 0.9552 (Table 5 and Figure 3).

**Table 5:** AUC analysis of circRMRP and circRPL27 in serum

circRNAs	AUC	95% CI	Sensitivity (%)	Specificity (%)	Maximum Yoden index
circRMRP	0.8646	0.7924–0.9368	82	22	0.60
circRPL27	0.9552	0.9103–1.000	94	8	0.86

## 4 Discussion

COPD is a preventable and treatable disease. Developing countries have high rates of COPD because of high prevalence of smoking. However, biomass fuel burning causes air pollution that also contributes to disease development [13]. Therefore, it is very important to diagnose COPD patients early and explore new diagnostic biomarkers and molecular targets to reveal the mechanism of COPD [14].

CircRNAs are characterized by structural stability, good conservation, and tissue specificity, which make them ideal molecules for prognostic biomarkers [15–17]. CircRNAs are crucial in the development of pulmonary conditions and are possible to become new diagnostic biomarkers and therapeutic targets for related diseases [18–20]. As an illustration, circFOXO3 is crucial in pathological inflammatory mechanisms [21]. Circ_0061052 controls miR-515-5p via the FoxC1/Snail regulatory pathway, playing a role in the epithelial–mesenchymal transformation and airway restructuring triggered by CSE [22]. Moreover, circRNAs may be involved in COPD by influencing immune homeostasis [11]. With the rapid development of gene sequencing technology, some studies have identified dysregulated circRNAs in COPD, mainly based on animal experiments and public databases. However, there is still a lack of research based on clinical samples.

It is worth noting that circRMRP and circRPL27 are known to be dysregulated in COPD and may have a potential role. However, so far, circRMRP and circRPL27 have not been reported in any COPD studies, so it is urgent to conduct more research on the mechanism of these two circRNAs in COPD to better understand their role in COPD. The results of RT-qPCR experiments showed that serum circRMRP and circRPL27 in COPD were elevated. Subsequently, RT-qPCR detected circRMRP and circRPL27 levels in the serum of three groups of COPD patients, namely, mild, moderate, and severe, respectively. The results noted that serum circRMRP and circRPL27 were higher in all three groups of COPD patients than in healthy controls, and their expression increased with the severity of COPD. Also circRMRP and circRPL27 were associated with smoking history, WBC, and FEV1/FVC. There is evidence that long-term exposure to cigarette smoke is the most important risk factor in the development of COPD [23]. Zeng et al. found that cigarette smoke significantly affected the expression of circRNAs, and pointed out that the differential expression patterns of circRNAs may be related to COPD caused by inhalation of smoke [24]. This suggests that the disorder of circRNA may be closely related to the inhalation of cigarette smoke. Long-term inhalation of cigarette smoke not only promotes the occurrence and development of COPD, but also further promotes the differential expression of circRNAs. This conjecture also needs further exploration in the future. In addition, circRMRP and circRPL27 were negatively correlated with FEV1/FVC, and circRPL27 was negatively correlated with FEV1, further suggesting that circRMRP and circRPL27 may affect lung status through lung function indicators, thereby participating in the progression of COPD [25,26].

In COPD, clinical evaluation and standard laboratory tests are often used to monitor the condition and have limited ability to predict disease progression and assess disease progression at an early stage. Therefore, it is particularly important to explore sensitive and non-invasive biomarkers for optimizing the early diagnosis and prognosis evaluation of patients with acute exacerbation of COPD. Therefore, we further evaluated the predictive efficacy of circRMRP and circRPL27 by drawing ROC curves. The results showed that circRMRP diagnosed COPD with a sensitivity of 82% and a specificity of 22%. CircRPL27 distinguished healthy subjects from COPD patients with 94% sensitivity and 8% specificity. These results indicate that circRMRP and circRPL27 have good predictive value for the diagnosis of COPD, and the diagnostic value of circRPL27 is better than that of circRMRP.

Due to the small number of participants in this study, additional validation of its findings is necessary in a larger group of COPD patients. Furthermore, RT-qPCR was the only method we used for assessing circRNA activity in COPD, which required overexpression or knockdown experiments to determine their role.

In conclusion, this study demonstrates the potential value of circRMRP and circRPL27 as non-invasive biomarkers for COPD testing.
